# Reliability of large language models in managing odontogenic sinusitis clinical scenarios: a preliminary multidisciplinary evaluation

**DOI:** 10.1007/s00405-023-08372-4

**Published:** 2024-01-08

**Authors:** Alberto Maria Saibene, Fabiana Allevi, Christian Calvo-Henriquez, Antonino Maniaci, Miguel Mayo-Yáñez, Alberto Paderno, Luigi Angelo Vaira, Giovanni Felisati, John R. Craig

**Affiliations:** 1 Otolaryngology Unit, Santi Paolo E Carlo Hospital, Department of Health Sciences, Università Degli Studi Di Milano, Milan, Italy; 2 Maxillofacial Surgery Unit, Santi Paolo E Carlo Hospital, Department of Health Sciences, Università Degli Studi Di Milano, Milan, Italy; 3 Service of Otolaryngology, Rhinology Unit, Hospital Complex at the University of Santiago de Compostela, Santiago de Compostela, A Coruña, Spain; 4 Department of Medical, Surgical Sciences and Advanced Technologies G.F. Ingrassia, University of Catania, Catania, Italy; 5 Otorhinolaryngology, Head and Neck Surgery Department, Complexo Hospitalario Universitario A Coruña (CHUAC), A Coruña, Galicia Spain; 6 Department of Otorhinolaryngology, Head and Neck Surgery, University of Brescia, Brescia, Italy; 7 Maxillofacial Surgery Operative Unit, Department of Medicine, Surgery and Pharmacy, University of Sassari, Sassari, Italy; 8 Biomedical Science PhD School, Biomedical Science Department, University of Sassari, Sassari, Italy; 9 Department of Otolaryngology-Head and Neck Surgery, Henry Ford Health, Detroit, MI USA

**Keywords:** Chronic rhinosinusitis, Maxillary sinusitis, Oroantral fistula, Dental implant, Computer-assisted diagnosis, Artificial intelligence

## Abstract

**Purpose:**

This study aimed to evaluate the utility of large language model (LLM) artificial intelligence tools, Chat Generative Pre-Trained Transformer (ChatGPT) versions 3.5 and 4, in managing complex otolaryngological clinical scenarios, specifically for the multidisciplinary management of odontogenic sinusitis (ODS).

**Methods:**

A prospective, structured multidisciplinary specialist evaluation was conducted using five ad hoc designed ODS-related clinical scenarios. LLM responses to these scenarios were critically reviewed by a multidisciplinary panel of eight specialist evaluators (2 ODS experts, 2 rhinologists, 2 general otolaryngologists, and 2 maxillofacial surgeons). Based on the level of disagreement from panel members, a Total Disagreement Score (TDS) was calculated for each LLM response, and TDS comparisons were made between ChatGPT3.5 and ChatGPT4, as well as between different evaluators.

**Results:**

While disagreement to some degree was demonstrated in 73/80 evaluator reviews of LLMs’ responses, TDSs were significantly lower for ChatGPT4 compared to ChatGPT3.5. Highest TDSs were found in the case of complicated ODS with orbital abscess, presumably due to increased case complexity with dental, rhinologic, and orbital factors affecting diagnostic and therapeutic options. There were no statistically significant differences in TDSs between evaluators’ specialties, though ODS experts and maxillofacial surgeons tended to assign higher TDSs.

**Conclusions:**

LLMs like ChatGPT, especially newer versions, showed potential for complimenting evidence-based clinical decision-making, but substantial disagreement was still demonstrated between LLMs and clinical specialists across most case examples, suggesting they are not yet optimal in aiding clinical management decisions. Future studies will be important to analyze LLMs’ performance as they evolve over time.

**Supplementary Information:**

The online version contains supplementary material available at 10.1007/s00405-023-08372-4.

## Introduction

Chat Generative Pre-Trained Transformer (ChatGPT, Open AI, San Francisco, CA, US) is the best-known example of a large language model (LLM), a text-interactive artificial intelligence (AI) trained on a wide range of texts available on the Internet. ChatGPT was trained with publicly available data sets consisting mostly of the Common Crawl (a publicly available data set of web pages), a data set of books and articles sourced from Project Gutenberg and other open-source data sets, and English Wikipedia pages (ref https://aboutchatgpt.com/data-source-of-chatgpt/). ChatGPT versions 3.5 and 4 were developed as easily accessible and user-friendly AI tools and have gained significant media attention due to their ability to interact textually with near-human capability [1].

Their apparent ease of use and unlimited capabilities draw attention and concerns from the healthcare community. Still, their role and potential limitations in healthcare have yet to be explored extensively, particularly in more niche settings such as Otolaryngology [2].

To explore the possibilities offered by LLM in managing complex otolaryngological scenarios, odontogenic sinusitis (ODS) represents an important and novel subject. ODS is a controversial multidisciplinary condition [3–5], whose diagnosis has been only recently addressed by international consensus [6] and has yet to be a topic of rhinologic AI research [7].

This study was designed to evaluate whether LLM can be helpful in managing niche clinical scenarios, by submitting to ChatGPT five ad hoc designed ODS-related cases and having a multidisciplinary panel analyze the AI replies. Other than simply testing ChatGPT 3.5 and 4 clinical management support capabilities, we aimed to determine whether the newer ChatGPT versions offered more reliable replies and whether different specialists reacted differently to the AI-generated replies.

## Methods

This study did not involve human participants, their data, or biological material. Therefore, it did not require institutional research ethics committee evaluation.

This study was designed as a prospective and structured multidisciplinary specialist evaluation of LLM management suggestions for four ODS cases and one case of unilateral rhinosinusitis that could mimic ODS. ODS diagnoses were defined by a recent international consensus statement by Craig et al. [6].

A single author (AMS) prepared the five text clinical cases. Three cases were designed to cover three groups of ODS etiologies[8] (case 1, right ODS due to apical periodontitis with right maxillary and ethmoid involvement; case 2, left ODS due to peri-implantitis with left maxillary, ethmoid, and frontal involvement; case 3, left ODS following maxillary sinus grafting with pan-sinus involvement and an adjacent orbital abscess). Case 4 depicted a recurrence of previously undiagnosed right ODS following root canal treatment. Case 5 depicted a non-sinusitis case with computed tomography of mild mucosal thickening around stable dental implants, thus mimicking ODS. The five cases were submitted to ChatGPT 3.5 and 4 on May 1, 2023 (available at https://openai.com/blog/chatgpt from OpenAI), with the detailed prompts reported in Online Resource 1, describing nasal endoscopy signs, patients' symptoms, and radiological reports and requesting the LLM to act as an otolaryngologist and correctly manage the patient.

The replies generated by each LLM were collected in a Google Documents file (Google LLC, Mountain View, California, US) and sent to the evaluation group. The evaluation group was composed of different specialists, as defined by their scientific output, including two ODS expert rhinologists (GF and JRC), two rhinologists (AM and CCH), two non-rhinologist otolaryngologists (MM and AP, whose research work is usually focused on head and neck surgery and oncology), and two maxillofacial surgeons (FA and LAV). The evaluation group members were informed that the replies they received were LLM-generated and revolved around ODS. The evaluation group was provided with a Google Sheets file (Google LLC), in which they were instructed to provide critical comments for each case and LLM reply concerning diagnosis, medical management, and surgical treatment, plus any other concerns that arose.

Answers for each domain (diagnosis, medical management, surgical treatment, other concerns) were scored on a four-point scale according to the degree of disagreement. The scale was as follows:

0, no disagreement.

1, minor disagreement (the answer was missing a non-critical detail).

2, moderate disagreement (one or more answer details were wrong, though they were not critical for the patient outcome).

3, major disagreement (the answer was lacking or wrongly reporting information that might be crucial for the patient outcome).

As the eight evaluators' were instructed to criticize the LLM output with textual responses, the degree of disagreement was scored separately by two authors according to the aforementioned scale. Any differences in scores were settled by consensus between evaluators. Evaluators’ critical commentaries were directed to the four defined domains (diagnosis, medical management, surgical treatment, other concerns), each one being scored separately for disagreement. The resulting scores for each domain were added to generate a total disagreement score (TDS) for each evaluator and LLM reply. Therefore, for each evaluator and LLM reply, the TDS might range from 0 (complete agreement in all four domains) to 12 (major disagreement in all four domains).

TDSs for each case were considered non-parametric data. Therefore, median and interquartile range (IQR) (reported as median[IQR]) were used as descriptive statistics for continuous data. Median TDS from both evaluations of any single case were compared between ChatGPT 3.5 and ChatGPT 4 with a Wilcoxon signed-rank test. Median TDSs were compared between each of the four groups of evaluators with a Kruskal–Wallis test. All statistical tests were performed using SPSS v. 28 (IBM Corp, Armonk, New York, US).

## Results

Answers generated by the two LLMs were reported following the prompt in Online Resource 1 (prompts in bold font, ChatGPT 3.5 replies in plain font, ChatGPT 4 replies in italics).

TDSs for each evaluator and Chat GPT answers are reported in Table 1, While Online Resource 2 reports the score for each domain. Case 5 (ODS mimic) received the lowest TDSs for both ChatGPT 3.5 (3[1.75]) and ChatGPT4 (2.5[1.25]), though ChatGPT 4 showed a similarly low TDS for ODS case 2 (2.5[3.25]). Note that for case 2, it was the only time ChatGPT 4 received a TDS of 0 from multiple evaluators. Case 3 (complicated ODS) received the highest TDSs for both ChatGPT 3.5 (8[2.5]) and ChatGPT 4 (4[2.25]). Two ChatGPT 3.5 answers and five ChatGPT 4 answers had TDSs of 0 when compared with some of the evaluators, though 73/80 responses suffered from some degree of disagreement. Major disagreements with the LLM replies were noted for 22 subitems in ChatGPT 3.5 answers, and only for 2 items in ChatGPT 4. The highest rate of major disagreements was found in the diagnostic domain (11 items), followed by medical and surgical management, while it was rarely reported for the “other concerns” domain. TDS was significantly lower for ChatGPT4 answers (3[2]) compared to ChatGPT3.5 answers (5[3], *p* < 0.001).

**Table 1:** Total disagreement scores for each answer and evaluator, (GPT3, Chat GPT 3.5 answer; GPT4, Chat GPT 4 answer; FA, LAV, MMY, AP, CCH, AM, GF, JRC, evaluators’ initials; TDS, total disagreement score; IQR, interquartile range)

	FA	LAV	MMY	AP	CCH	AM	GF	JRC	Overall TDS	Median TDS	TDS IQR	Minimum TDS	Maximum TDS
Case 1													
GPT3	5	6	4	5	3	5	5	6	39	5	0.5	3	6
GPT4	3	2	4	3	4	3	3	6	28	3	1	2	6
Case 2													
GPT3	4	6	4	5	4	3	5	4	35	4	1	3	6
GPT4	0	2	4	3	0	3	0	4	16	2.5	3.25	0	4
Case 3													
GPT3	6	8	5	8	8	3	9	9	56	8	2.5	3	9
GPT4	4	2	2	4	5	5	3	5	30	4	2.25	2	5
Case 4													
GPT3	6	6	2	3	0	3	6	7	33	4.5	3.25	0	7
GPT4	4	3	2	3	0	3	2	4	21	3	1.25	0	4
Case 5													
GPT3	0	5	1	3	3	3	4	7	26	3	1.75	0	7
GPT4	0	3	1	2	4	2	3	3	18	2.5	1.25	0	4

**Fig. 1:**
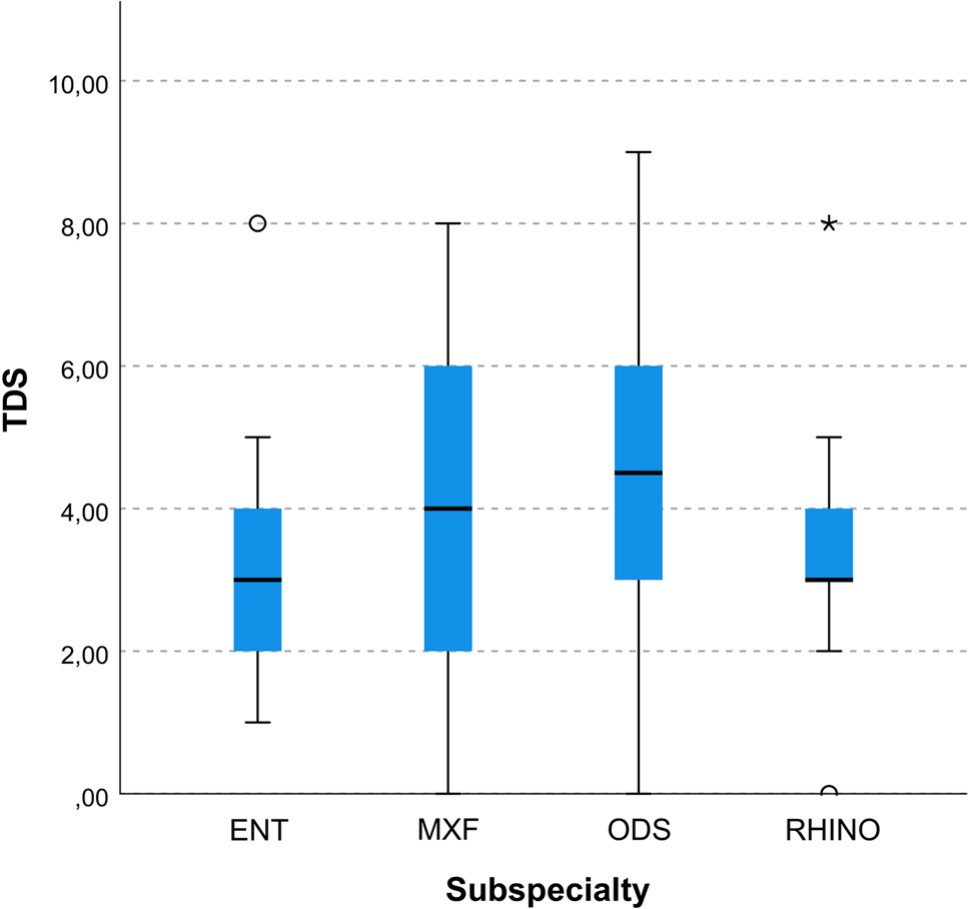
Box and whiskers plot showing the distribution of total disagreement scores (TDS) according to the subspecialty of evaluators (*ENT* non-rhinology otolaryngologists, *MXF* maxillofacial surgeons, *ODS* odontogenic sinusitis specialists, *RHINO* rhinologists)

Figure 1 shows the median TDSs for all answers according to each group of evaluators. There were no statistically significant differences in TDSs based on raters’ specialty groupings, although there was a tendency towards higher TDSs with maxillofacial surgeons (4[4]) and ODS experts (4.5[3]), *p* = 0.085. Maxillofacial surgeons and ODS experts showed a general tendency towards a stronger criticism of the LLM answers, which was not limited to specific domains or cases.

## Discussion

The application of LLMs in the medical field is a rapidly evolving area, with the potential to aid in clinical decision-making, especially in subspecialty fields [9, 10]. Yet, any evaluation of these tools must be context-specific and rigorous. This assessment is particularly relevant, urgent, and novel for complex conditions, such as ODS, where optimal multidisciplinary diagnostic and therapeutic paradigms are challenging to establish due to a relatively scarce body of recently published evidence [11]. This study aimed to evaluate the reliability of two versions of the ChatGPT LLM in managing ODS. To the authors’ knowledge, this was the first study to assess systematically the ability of LLM to manage niche multidisciplinary clinical scenarios, specifically ODS.

The results highlighted several areas, where the LLMs’ performances were suboptimal. These limitations ranged from minor disagreements to more critical discrepancies in the responses given by the LLMs, suggesting that the current versions would not be reliable as standalone decision-making tools for ODS. Diagnosing and managing ODS can be challenging, requiring a nuanced understanding of its pathogenesis, a broad array of causative etiologies, and frequent overlap of non-specific symptoms which make it difficult to distinguish from other sinonasal conditions. It would seem that LLMs could not consistently identify and process some of these nuances. For example, major disagreements were especially noted by all evaluators in the diagnosis domains of LLM responses. LLMs may not have consistently understood the potential pathogenic connection between dental procedures, dental conditions, and sinusitis. LLMs also did not use consensus-based ODS definitions, diagnostic classifications, or acronyms as proposed either by Felisati et al. or Craig et al. [6, 8, 12, 13]. Chat GPT 3.5 even failed to use the word “odontogenic”, while Chat GPT4 did use this diagnostic term. Higher disagreement in this domain could also be due to the perceived need for a “precise” diagnosis, which led to stronger criticism of vague LLM replies. Another area of concern was illustrated by the significantly higher TDSs for both LLM versions when evaluating the complicated ODS case (Case 3). For example, ChatGPT 3.5 failed to understand the potential for ODS to cause extra-sinus orbital, intracranial, or osseous infectious complications, and ChatGPT4 still failed to prioritize emergency interventions adequately when compared to the literature [14]. LLM performance was relatively better in less complex cases, such as case 2 (overt ODS) as well as case 5 (rhinosinusitis mimicking ODS). This suggests that the LLMs may have some utility in ruling in ODS when the presentation is classic, and ruling out ODS when ODS likelihood is low. The lower rate of TDS for medical and surgical management, on the other hand, might be due to the multiple therapeutic options that can be proposed to ODS patients firsthand, thus giving LLMs a larger interpretation margin. In these regards, it is indeed interesting that LLMs did not provide several management options for each case, but they did demonstrate some flexibility in proposing composite management plans that might discretionally include two or more options (combined or not). Thus ChatGPT appears limited in helping select a specific treatment plan but does provide a rather comprehensive—though often convolute—overview of available options.

Another important point to highlight is that while TDSs varied substantially across different case types, with most responses generating some degree of disagreement, Chat GPT4 clearly outperformed ChatGPT3.5 with lower TDSs, presumably due to its higher number of parameters and improved architecture (though precise training data sets are not publicly available). The lower TDSs often resulted from longer answers covering more differential diagnoses or treatment possibilities, but choice prioritization was not always clear. This finding highlights an intrinsic limitation of LLMs; they are ultra-wide encyclopedic references incapable of clinical reasoning [15] and have not yet reached the reasoning potential of general artificial intelligence [7]. However, these results also bring to light a very exciting implication with regard to facilitating multidisciplinary management of complex conditions, for which published evidence may be relatively new, and yet to be highlighted in specialty guidelines. ODS is a great example of this since more attention has been placed on researching the entity only recently. LLMs and other AI technologies could potentially obtain such newly published information immediately if available online. If such AI technologies reached appropriate clinical accuracy, clinicians could call on the most up-to-date evidence instantaneously, saving a great deal of time for clinicians and providing patients with the highest quality treatment options. While this implication is exciting, AI tools must evolve to reach a point where online information and published literature are prioritized in a way to optimize clinical utility. In these regards, training LLMs only on publicly available data sets could induce another bias in replies by omitting potentially important recent scientific data protected by a paywall. Furthermore, some researchers have theorized potential changes over time in the performance of the LLMs analyzed in this study [16], and if true, findings from this study highlight the need for regular assessments of LLM performance metrics to ensure appropriate use in clinical practice.

However, the clinical field is characterized by specific needs and requirements that may not be suited for general-purpose LLMs. To this end, some researchers have tried developing AI models fine-tuned for the medical domain. For example, Med Palm 2 by Google Research has shown remarkable results in answering medical questions with striking improvements when compared with its previous iteration, hinting at a promising path for future research [17].

When developing performance metrics for these clinical LLMs, it will be important to reach a consensus between experts in the given field of interest. As an example, while not reaching statistical significance in this small preliminary study, maxillofacial surgeons and ODS experts assigned higher TDSs to LLM responses. On the one hand, this discrepancy reflects the inherent subjectivity when interpreting AI-generated responses, further emphasizing the need for validated measures to evaluate AI tools. On the other hand, it highlights that ODS experts and maxillofacial surgeons may be more attuned to the nuances of managing ODS. Again, these specialists might simply tend to be more vocal in expressing their criticism in a research field they feel closer to their day-to-day clinical routine. For example, clinicians should be aware of both the distinct inflammatory [18] and infectious sinusitis [19, 20] as well as the numerous odontogenic]or dental treatment-related causes of ODS [6, 21]. Since ODS has not been highlighted adequately in otolaryngologic or dental therapeutic guidelines, non-ODS experts may not be aware of some of these diagnostic and therapeutic nuances [6, 22, 23]. These factors should be considered when developing validated measures of accuracy for LLMs and other AI technologies being utilized for clinical decision-making.

When considering the LLM analysis, it has to be noted that in this case, we chose to provide the AI with strictly medical information, trying to be as objective as possible, both through complete data reporting and language clarity. Whether an LLM would be able to interpret a clinical scenario through lay terms patient-reported signs and symptoms and clinical/radiological pictures (through its recent Chat-GPT 4 V evolution) should be subjected to further specific analysis. Such an analysis—albeit interesting—would work on a double operational level for the LLM, as the lay language analysis and clinical picture interpretation represent a further potential confounding factor. As little is known about the potential of these AI tools, we opted in this work to minimize the bias focusing only on exploring the capabilities in terms of clinical management.

This small preliminary study had indeed several limitations. First, analyzing more clinical cases with a broader group of evaluators could have led to identifying more specific behavioral patterns for LLMs, highlighting other potential strengths and weaknesses. Second, potential bias was introduced since evaluators were aware of the focus on ODS. This bias could be avoided by mixing ODS cases with non-odontogenic sinus disease cases. Future studies can build on this study by generating a larger set of all rhinologic conditions. Finally, while the proposed TDS was an attempt at generating a homogeneous and objective interpretation of AI and clinician evaluations, this system has not been validated and, therefore, should be viewed with caution. In these regards, though other LLM output scorings have been proposed while the present research was ongoing [24], TDS may represent a swift tool for testing AI interpretations over a significant number of cases and with multiple operators. This is due to TDS not requiring training each rater, as its value can be calculated by having one or—preferably two—researchers rating textual replies in the single domains, which, in turn, can be adapted to most clinical scenarios. It is indeed of the utmost importance that future research be dedicated to developing validated scoring systems to analyze LLMs in a more reproducible fashion across research studies.

As it would be even more interesting to study the tendencies of LLM behavior for diagnostic and therapeutic purposes in different niche and non-niche settings over a more consistent number of raters, the development of strong LLM evaluation tools, TDM being one of them, is in our opinion pivotal.

## Conclusion

While LLMs such as ChatGPT, especially newer versions, offer significant potential in complimenting evidence-based clinical decision-making, the substantial variability in TDSs across case examples in this study suggests that they are not yet optimal for aiding clinical management. Future studies will be important for analyzing LLMs’ performance as they evolve over time.

### Supplementary Information

Below is the link to the electronic supplementary material.

Supplementary file1 (DOCX 23 KB)

Supplementary file2 (XLSX 36 KB)

## Data Availability

All data pertaining to this systematic review are available from the corresponding author upon reasonable request.
