# Supplementary material for: Reliability of large language models in managing odontogenic sinusitis clinical scenarios: a preliminary multidisciplinary evaluation
Source: Eur Arch Otorhinolaryngol. 2024 Jan 8;281(4):1835–41. doi: 10.1007/s00405-023-08372-4 (PMC10943141; doi:10.1007/s00405-023-08372-4)
Supplement: Supplementary file 1 — Supplementary file1 (DOCX 23 KB) [file 405_2023_8372_MOESM1_ESM.docx]

***Article title: Reliability of large language models in managing odontogenic sinusitis clinical scenarios: a preliminary multidisciplinary evaluation***

***Journal: European Archives of Oto-Rhino-Laryngology***

***Authors, affiliation and emails: blinded for peer-reviewing purposes***

**CASE 1**

**The setting is an inpatient otorhinolaryngological clinic. The otolaryngologist is examining for the first time a patient, a 42 years old male with no other significant medical history. The patient has been experiencing right facial pain, foul nasal discharge and nasal obstruction for 3 months. The computed tomography shows complete opacification of the right maxillary and ethmoid sinus with cortical bone focal erosion over the second upper right molar. The endoscopy shows purulent discharge in the right nasal cavity. What would the otolaryngologist think about the diagnosis? How would the doctor manage the patient from a clinical and surgical point of view? Provide detailed diagnosis and - if needed - drugs and surgical procedures the otorhinolaryngologist would employ in this case.**

Based on the patient's symptoms and the imaging findings, the otolaryngologist would most likely suspect that the patient has chronic rhinosinusitis with associated maxillary and ethmoid sinusitis. The focal erosion of the cortical bone over the second upper right molar suggests that the infection may have spread from the sinus to the adjacent tooth.

The otolaryngologist would first perform a thorough evaluation of the patient's medical history and a physical examination of the head and neck. This would include a detailed examination of the nasal cavity using an endoscope to confirm the presence of purulent discharge and to assess the extent of the infection.

The otolaryngologist would then order further imaging studies to confirm the diagnosis and to determine the extent of the disease. This may include a magnetic resonance imaging (MRI) scan, a computed tomography (CT) scan, or both.

Once the diagnosis is confirmed, the otolaryngologist would likely recommend a combination of medical and surgical management. The medical management may include antibiotics, nasal corticosteroids, and saline nasal irrigation to reduce inflammation and promote drainage of the sinuses.

If the patient's symptoms do not improve with medical management, or if the infection has spread to adjacent structures such as the teeth or the eye, the otolaryngologist may recommend surgical intervention. This may involve endoscopic sinus surgery to remove any obstructive tissue, to provide better drainage of the sinuses, and to promote healing of the infected tissue.

In addition to endoscopic sinus surgery, the otolaryngologist may also recommend a dental consultation to address the focal erosion over the second upper right molar. The dental consultation may result in a root canal treatment or tooth extraction as needed.

Overall, the treatment plan for this patient with chronic rhinosinusitis and associated maxillary and ethmoid sinusitis would depend on the severity of the disease and the extent of the infection. The otolaryngologist would work closely with other specialists as needed to provide comprehensive care for the patient.

*Based on the patient's symptoms, imaging, and endoscopic findings, the otolaryngologist would likely consider a diagnosis of chronic rhinosinusitis with possible odontogenic origin. The chronic facial pain, nasal obstruction, foul discharge, and imaging findings of opacification and bone erosion all support this diagnosis.*

***Diagnosis:***

*1. Chronic rhinosinusitis: Inflammation of the nasal passages and paranasal sinuses lasting more than 12 weeks. The patient's symptoms and imaging findings are consistent with this diagnosis.*

*2. Possible odontogenic origin: The focal erosion of the cortical bone over the second upper right molar may indicate that the sinusitis has an odontogenic origin, possibly from a dental infection or dental procedure complication.*

***Clinical Management:***

*Before considering surgery, the patient would be managed conservatively with medicaltreatment. This may include:*

*1. Antibiotics: A broad-spectrum oral antibiotic such as amoxicillin-clavulanate (e.g., Augmentin) for 2-4 weeks to target the likely bacterial pathogens causing the infection. If the patient has a penicillin allergy, alternatives like clindamycin or a respiratory fluoroquinolone (e.g., levofloxacin) could be considered.*

*2. Nasal corticosteroids: Intranasal corticosteroids like fluticasone or mometasone can help reduce inflammation and improve symptoms.*

*3. Nasal saline irrigation: Saline nasal rinses can help clear mucus and improve sinus drainage.*

*4. Decongestants: Oral or topical decongestants (e.g., pseudoephedrine, oxymetazoline) can provide temporary relief of nasal congestion but should be used cautiously, as overuse may worsen symptoms.*

*5. Pain management: Over-the-counter analgesics like acetaminophen or ibuprofen can help relieve facial pain.*

*6. Dental evaluation: Referral to a dentist or an oral surgeon for evaluation of the second upper right molar and possible dental treatment if there is an odontogenic source for the sinusitis.*

***Surgical Management:***

*If conservative medical management fails, or if there are complications such as recurrent or persistent infections, the otolaryngologist may consider the following surgical options:*

*1. Functional endoscopic sinus surgery (FESS): This minimally invasive procedure involves the use of an endoscope to remove obstructive or infected tissue and improve sinus drainage. For this patient, FESS could involve opening the maxillary and ethmoid sinuses to facilitate drainage and removal of infected material.*

*2. Caldwell-Luc procedure: If FESS is not sufficient, or if the odontogenic source is confirmed, the Caldwell-Luc procedure may be considered. This surgical approach involves creating an opening in the maxillary sinus via the oral cavity and removing the infected tissue. It may also involve treating the dental infection and/or extracting the affected tooth.*

*3. Other procedures: Depending on the specific situation, other surgical interventions such as septoplasty (to correct a deviated nasal septum) or turbinate reduction (to improve nasal airflow) may be considered.*

*The otolaryngologist would tailor the management plan according to the patient's individual needs, response to treatment, and any identified underlying causes. The ultimate goal is to resolve the infection, improve sinus drainage, and alleviate the patient's symptoms.*

**CASE 2**

**The setting is an inpatient otorhinolaryngological clinic. The otolaryngologist is examining for the first time a patient, a 55 years old female with known type 2 diabetes and hypertension. The patient has been referred by her dentist, who discovered a left maxillary sinus opacification while performing a cone beam computed tomography for implant placement in the right maxillary alveolar ridge. The patient, who had dental implants placed in the left maxillary alveolar ridge 2 years before, has already undergone a head computed tomography. This scan shows complete opacification of the left maxillary, frontal, and ethmoid sinus. The first cone beam computed tomography also showed bony rarefaction around one of the left side implants. The endoscopy shows purulent discharge in the right nasal cavity, and uncinate process edema. What would the otolaryngologist think about the diagnosis? How would the doctor manage the patient from a clinical and surgical point of view? Provide detailed diagnosis and - if needed - drugs and surgical procedures the otorhinolaryngologist would employ in this case.**

Based on the patient's history, imaging studies, and physical examination findings, the otolaryngologist would likely suspect that the patient has chronic sinusitis with possible peri-implantitis in the left maxillary sinus. Chronic sinusitis is a common inflammatory condition of the paranasal sinuses that can lead to recurrent infections and persistent inflammation. Peri-implantitis is inflammation and infection around a dental implant, which can lead to implant loss if left untreated.

The opacification of the left maxillary, frontal, and ethmoid sinus on the head CT scan is indicative of chronic sinusitis. The rarefaction around one of the left side implants seen on the cone beam CT scan suggests peri-implantitis. The presence of purulent discharge in the right nasal cavity and uncinate process edema on endoscopy are also consistent with chronic sinusitis.

The otolaryngologist would likely manage the patient with a combination of medical and surgical interventions. Medical management would involve antibiotics and nasal saline irrigation to treat the infection and reduce inflammation. The choice of antibiotics would depend on the severity of the infection and the patient's medical history. In this case, the patient has type 2 diabetes and hypertension, which may affect the choice of antibiotics. The otolaryngologist may consult with the patient's primary care physician or endocrinologist to ensure that the chosen antibiotics are safe and effective for the patient.

Surgical management would involve endoscopic sinus surgery (ESS) to remove any obstructing tissue and to open the sinuses for better drainage. The ESS would likely involve the removal of the uncinate process, which is a bony projection that obstructs the drainage pathway of the maxillary sinus. The ESS would also allow for a better assessment of the peri-implantitis around the left maxillary implant. The implant may need to be removed if the peri-implantitis is severe, and the implant is deemed non-salvageable. If the implant can be saved, it may require debridement and antibiotics to treat the infection.

In summary, the otolaryngologist would diagnose the patient with chronic sinusitis with possible peri-implantitis in the left maxillary sinus. The treatment would involve a combination of medical and surgical interventions, including antibiotics, nasal saline irrigation, and endoscopic sinus surgery. The surgical procedure would likely involve the removal of the uncinate process and assessment of the peri-implantitis around the left maxillary implant, which may require implant removal or debridement and antibiotics. The management of this patient would require a multidisciplinary approach, involving the otolaryngologist, the patient's primary care physician, and potentially an endocrinologist.

*Based on the provided information, the otolaryngologist might suspect the patient has chronic sinusitis, possibly secondary to or exacerbated by the dental implant. The complete opacification of the left maxillary, frontal, and ethmoid sinus, bony rarefaction around the dental implant, and purulent discharge in the right nasal cavity are all indicative of sinusitis. Additionally, the patient's underlying conditions, such as type 2 diabetes and hypertension, may contribute to an increased risk of infection and complications.*

***Diagnosis:***

*1. Chronic sinusitis (possibly odontogenic in origin, related to the dental implant).*

*2. Uncinate process edema.*

***Management:***

*The management of this case would involve both medical and surgical approaches, as follows:*

***Medical management:***

*1. Broad-spectrum antibiotics: The otolaryngologist may prescribe oral antibiotics to treat the sinus infection. The choice of antibiotic could depend on local resistance patterns, but options might include amoxicillin-clavulanate, levofloxacin, or moxifloxacin. Treatment duration may be 3-4 weeks or longer, depending on the clinical response.*

*2. Nasal corticosteroids: These medications can help decrease inflammation and promote sinus drainage. Examples include fluticasone or mometasone nasal spray.*

*3. Nasal saline irrigation: The patient might be advised to perform regular saline irrigation to help clear out mucus and reduce inflammation.*

*4. Pain management: Over-the-counter pain relievers like acetaminophen or ibuprofen may be recommended to help manage pain and discomfort associated with sinusitis.*

*5. Control of underlying conditions: The patient should continue managing her type 2 diabetes and hypertension as advised by her primary care physician.*

***Surgical management:***

*If medical management fails to improve the patient's symptoms or if there is concern about the dental implant being the source of the infection, surgical intervention may be considered.*

*1. Functional endoscopic sinus surgery (FESS): This minimally invasive procedure involves the use of an endoscope to remove any obstructive tissue, polyps, or diseased mucosa from the sinus cavities, improving drainage and ventilation. The specific techniques employed may vary depending on the patient's sinus anatomy and the extent of the disease.*

*2. Dental implant evaluation and possible removal: The otolaryngologist may consult with the patient's dentist or an oral and maxillofacial surgeon to evaluate the dental implant's role in the infection. If the implant is found to be the source of the infection, removal of the implant and appropriate treatment of the affected area may be necessary.*

*Close follow-up and monitoring of the patient's condition will be essential for successful management. If the patient does not respond to initial treatments or if her condition worsens, further evaluation and management adjustments may be required.*

**CASE 3**

**The setting is an inpatient otorhinolaryngological clinic. The otolaryngologist is examining for the first time a patient, a 70 years old male with no other known comorbidity. The patient has been referred by the emergency department where he came due to a high fever (40°C), headache, left facial pain, left proptosis, left nasal discharge, and congestion. Nasal symptoms have been worsening for one week despite treatment with amoxicillin-clavulanate 1 g three times a day. Proptosis appeared during the last 24 hours. The patient underwent a left maxillary sinus grafting for future dental implant positioning one month before. The contrast-enhanced computed tomography performed in the emergency department shows complete opacification of the left maxillary sinus, ethmoid sinus, frontal sinus, and sphenoid sinus, a right wide septal deviation, hyperdense material dispersed in the left maxillary sinus and a left medial orbital abscess. The endoscopy shows purulent discharge in the left nasal cavity. Upon ophthalmological examination, the patient has reduced left-side visual acuity and loss of red color perception. What would the otolaryngologist think about the diagnosis? How would the doctor manage the patient from a clinical and surgical point of view? Provide detailed diagnosis and - if needed - drugs and surgical procedures the otorhinolaryngologist would employ in this case.**

Based on the patient's symptoms, history, and imaging findings, the otolaryngologist would suspect that the patient has acute invasive fungal sinusitis, also known as fungal rhinosinusitis. This is a rare but serious condition that often affects immunocompromised patients, but can also occur in immunocompetent individuals. It is characterized by the invasion of fungal hyphae into the mucosa and surrounding tissues of the sinuses and can spread to adjacent structures, such as the orbit and brain.

The presence of left facial pain, left proptosis, and left medial orbital abscess are suggestive of orbital involvement, which is a serious complication of fungal rhinosinusitis. The reduced left-side visual acuity and loss of red color perception indicates optic nerve involvement, which is also a serious complication.

The fact that the patient had undergone recent maxillary sinus grafting may have contributed to the development of fungal rhinosinusitis, as this procedure can disrupt the normal mucociliary clearance system of the sinuses and provide a favorable environment for fungal growth.

The otolaryngologist would first initiate broad-spectrum antifungal therapy, such as intravenous amphotericin B or voriconazole, to halt the progression of the infection. The patient may also require hospitalization for close monitoring and supportive care.

Surgical intervention is often necessary in cases of fungal rhinosinusitis, particularly if there is evidence of orbital or intracranial involvement. The otolaryngologist would typically perform a functional endoscopic sinus surgery (FESS) to remove diseased tissue and debris from the sinuses, and to provide access for topical antifungal therapy.

In cases of orbital involvement, the otolaryngologist may collaborate with an ophthalmologist to perform orbital decompression surgery to relieve pressure on the optic nerve and to allow for better drainage of the abscess.

The otolaryngologist would also closely monitor the patient's response to treatment and adjust the therapy as needed based on clinical and radiographic findings. The patient may require long-term antifungal therapy and close follow-up to ensure that the infection has been completely eradicated.

*The otolaryngologist would likely consider the following diagnosis:*

***Diagnosis:** Complicated acute rhinosinusitis with orbital cellulitis and abscess (potentially arising from a complication of the recent left maxillary sinus grafting procedure).*

*Given the patient's clinical presentation and imaging findings, the doctor would manage the patient both medically and surgically.*

***Medical management:***

*1. Intravenous broad-spectrum antibiotics: The patient should be started on IV antibiotics to cover a wide range of aerobic and anaerobic bacteria, considering the severity of the infection and the involvement of the orbit. A combination of vancomycin (or linezolid if there's a history of MRSA) and either ceftriaxone or piperacillin-tazobactam would be appropriate.*

*2. Corticosteroids: The use of systemic corticosteroids (e.g., prednisolone) can be considered to reduce inflammation and edema, as long as there's no contraindication.*

*3. Analgesia: Pain management with appropriate analgesic medications (e.g., paracetamol, NSAIDs) should be provided.*

*4. Antipyretics: To manage the high fever, antipyretic medications (e.g., paracetamol) can be given.*

*5. Fluids and electrolytes: Ensure adequate hydration and electrolyte balance.*

***Surgical management:***

*Considering the patient's severe symptoms, orbital involvement, and the presence of an orbital abscess, surgical intervention is likely necessary.*

*1. Endoscopic sinus surgery: The otolaryngologist would perform an endoscopic sinus surgery to drain the involved sinuses (e.g., left maxillary, ethmoid, frontal, and sphenoid sinuses). This may involve creating an opening in the maxillary sinus (maxillary antrostomy), ethmoidectomy, and possibly frontal sinus drainage (e.g., Draf IIa or Draf III procedure) and sphenoidotomy.*

*2. Orbital abscess drainage: In collaboration with an ophthalmologist or an oculoplastic surgeon, the otolaryngologist may also need to perform a drainage procedure for the left medial orbital abscess.*

*The patient should be closely monitored for improvement in symptoms and resolution of the infection. Regular ophthalmological evaluation should be done to assess the visual acuity and color perception.*

*In case the patient does not respond to the initial treatment, the otolaryngologist may need to re-evaluate the antibiotic regimen and consider the possibility of resistant organisms or fungal infection as a cause of the symptoms. Additionally, further surgical intervention may be necessary if there is no improvement or if complications arise.*

**CASE 4**

**The setting is an inpatient otorhinolaryngological clinic. The otolaryngologist is examining a patient who already underwent an endoscopic sinus surgery procedure (right middle antrostomy, complete ethmoidectomy, sphenoidotomy, and Draft IIb) a year before for chronic rhinosinusitis without nasal polyps affecting only the right side. The patient is a 35 years old female with a history of migraine with aura. The patient has been experiencing recurring right facial pain and nasal crusting starting three months after the endoscopic sinus surgery procedure, with only temporary improvement with oral antibiotics. The symptoms were worse before endoscopic sinus surgery. The computed tomography performed after the procedure shows mild opacification of the right maxillary sinus, especially at the bottom of the sinus, and a normal postoperative appearance of all other sinuses. The left sinonasal cavities are normal. Several upper left and right tooth show signs of endodontic procedures, though no bony erosion is shown. The endoscopy shows crusts and purulent content coming from the right maxillary sinus. There’s no sign of surgical complications, missed ostium sequence, synechiae, or other scars which might decrease the maxillary drainage. What would the otolaryngologist think about the diagnosis? How would the doctor manage the patient from a clinical and surgical point of view? Provide detailed diagnosis and - if needed - drugs and surgical procedures the otorhinolaryngologist would employ in this case.**

Based on the patient's history and symptoms, the otolaryngologist would likely suspect that the patient is experiencing recurrent sinusitis in the right maxillary sinus despite having undergone endoscopic sinus surgery. The CT scan findings of mild opacification of the right maxillary sinus at the bottom of the sinus would support this suspicion.

The otolaryngologist may consider several possible causes for the recurrent sinusitis, including inadequate drainage of the right maxillary sinus, persistent infection, or a complication of the previous surgery. The absence of any signs of surgical complications, missed ostium sequence, synechiae or other scars that might decrease the maxillary drainage would make it less likely that the patient is experiencing a complication of the previous surgery.

The otolaryngologist may consider performing a diagnostic nasal endoscopy to further evaluate the right maxillary sinus and to obtain cultures to identify the causative organism, if any. The presence of purulent content coming from the right maxillary sinus, as seen on the endoscopy, would support the diagnosis of recurrent sinusitis.

The otolaryngologist may initially manage the patient with a course of broad-spectrum antibiotics, such as amoxicillin-clavulanate or doxycycline, to target common bacterial pathogens that cause sinusitis. The patient's history of migraine with aura should be considered when selecting an appropriate antibiotic, as some antibiotics may increase the risk of migraine attacks.

If the patient fails to respond to antibiotics, the otolaryngologist may consider a repeat endoscopic sinus surgery to address any persistent drainage or infection in the right maxillary sinus. The surgical approach may include revision of the middle antrostomy, targeted debridement of the right maxillary sinus, or placement of a stent to maintain patency of the maxillary ostium.

In addition to antibiotics and surgery, the otolaryngologist may also recommend saline irrigation of the sinonasal cavities to help clear any mucus and debris that may be contributing to the patient's symptoms. The patient should also be advised to avoid any known triggers for migraine attacks and to manage any migraine symptoms with appropriate medications, as needed.

Overall, the otolaryngologist would diagnose the patient with recurrent sinusitis in the right maxillary sinus and would manage the patient with a combination of antibiotics, surgery, and supportive measures to address the underlying cause of the symptoms.

*The otolaryngologist may consider the following diagnoses in this case:*

*1. Recurrent or persistent chronic maxillary sinusitis on the right side, possibly due to ongoing infection or inflammation.*

*2. Maxillary sinusitis of dental origin, given the history of endodontic procedures on the upper teeth.*

*3. Migraine-related facial pain or neuralgia, considering the patient's history of migraine with aura.*

*To manage the patient, the otolaryngologist might take a stepwise approach:*

***Medical management:***

*1. *Antibiotics*: A course of broad-spectrum antibiotics, such as amoxicillin-clavulanate or a fluoroquinolone, may be prescribed to target the ongoing infection. The duration of the antibiotic course might vary from 2 to 4 weeks, depending on the severity of the symptoms and the patient's response to treatment.*

*2. *Topical nasal corticosteroids*: These can be used to reduce inflammation and improve nasal airflow, potentially aiding in the resolution of the sinus infection.*

*3. *Nasal saline irrigation*: This can help maintain nasal hygiene, reduce crusting, and improve mucociliary clearance.*

*4. *Pain management*: Over-the-counter analgesics, such as ibuprofen or acetaminophen, can be used to manage facial pain. If the pain is suspected to be migraine-related, specific migraine medications may be prescribed.*

*5. *Referral to a dentist*: If maxillary sinusitis of dental origin is a concern, the patient should be referred to a dentist for a thorough evaluation and treatment of any potential dental issues.*

***Surgical management:***

*If the patient fails to respond to medical management, the otolaryngologist may consider additional surgical interventions:*

*1. *Revision endoscopic sinus surgery*: This might involve a more extensive maxillary antrostomy or even a modified endoscopic medial maxillectomy to improve drainage and ventilation of the maxillary sinus.*

*2. *Functional endoscopic sinus surgery (FESS)*: If other sinuses are found to be involved, FESS can be considered to address the issue.*

*The otolaryngologist should closely monitor the patient's progress and response to treatment, adjusting the management plan as necessary. Collaboration with other specialists, such as dentists or neurologists, may be beneficial in managing this patient's complex symptoms.*

**CASE 5**

**The setting is an inpatient otorhinolaryngological clinic. The otolaryngologist is examining for the first time a patient, a 73 years old female with known hypertension, dyslipidemia, and allergic rhinitis. The patient has been referred by her dentist, who discovered a partial right maxillary sinus opacification while performing a cone beam computed tomography for implant placement in the right maxillary alveolar ridge. The patient complains of bilateral nasal obstruction dating back at least 20 years. The patient, who had other dental implants placed in the same maxillary alveolar ridge 2 years before, has already undergone a head computed tomography. This scan shows partial opacification of the right maxillary (about 10 mm in height, mostly at the bottom of the maxillary sinus) and right ethmoid sinus, bilateral inferior turbinate hypertrophy, and right septal deviation. There are no signs of bony rarefaction around the other dental implants. The endoscopy confirms turbinate hypertrophy and septal deviation, but there is no purulent discharge in the nasal cavities, no polyps, and no signs of malignancies, What would the otolaryngologist think about the diagnosis? How would the doctor manage the patient from a clinical and surgical point of view? Provide detailed diagnosis and - if needed - drugs and surgical procedures the otorhinolaryngologist would employ in this case.**

Based on the patient's history and examination findings, the otolaryngologist may suspect that the patient is suffering from chronic rhinosinusitis with nasal obstruction. The opacification of the maxillary and ethmoid sinuses seen on the head CT scan indicates chronic inflammation in these sinuses, which is a common finding in chronic rhinosinusitis. The patient's long-standing history of bilateral nasal obstruction also supports this diagnosis.

In addition, the patient has bilateral inferior turbinate hypertrophy and right septal deviation, which can contribute to nasal obstruction. The allergic rhinitis may also be a contributing factor. The absence of purulent discharge, polyps or malignancies on endoscopy suggests that the patient may have a non-infectious form of chronic rhinosinusitis.

The partial opacification of the right maxillary sinus seen on the dental cone beam CT scan may be unrelated to the chronic rhinosinusitis or may be a result of chronic inflammation in the sinus. However, given the absence of signs of infection or malignancy, the otolaryngologist may choose to monitor this finding and repeat imaging in the future to evaluate for any changes.

From a clinical point of view, the otolaryngologist may recommend medical therapy to manage the patient's chronic rhinosinusitis and nasal obstruction. This may include intranasal corticosteroids, saline irrigation, and antihistamines for the allergic rhinitis. The otolaryngologist may also recommend a trial of nasal steroid sprays and/or oral leukotriene modifiers to reduce the inferior turbinate hypertrophy.

If medical therapy fails to provide relief, the otolaryngologist may consider surgical intervention. The patient's right septal deviation may be corrected with a septoplasty, and the inferior turbinate hypertrophy may be addressed with a turbinoplasty. The otolaryngologist may also consider endoscopic sinus surgery to improve the ventilation and drainage of the sinuses.

In summary, the otolaryngologist may diagnose the patient with chronic rhinosinusitis with nasal obstruction, likely due to a combination of factors including chronic inflammation in the sinuses, inferior turbinate hypertrophy, and septal deviation. Medical therapy with intranasal corticosteroids, saline irrigation, and antihistamines may be recommended initially, with surgical intervention considered if medical therapy fails to provide relief.

*I am not a doctor, but based on the information provided, the otolaryngologist might consider the following diagnosis and management plan for the patient:*

***Diagnosis:***

*1. Chronic rhinosinusitis without nasal polyps (CRSsNP)*

*2. Bilateral inferior turbinate hypertrophy*

*3. Right septal deviation*

***Clinical management:***

*1. Prescribe a nasal corticosteroid spray (e.g., fluticasone or mometasone) to reduce inflammation in the nasal passages.*

*2. Recommend saline nasal irrigation to help clear mucus and improve nasal breathing.*

*3. Prescribe an intranasal antihistamine spray (e.g., azelastine) if the allergic component of the patient's rhinitis is significant and not well-controlled.*

*4. Advise the patient on environmental control measures to reduce exposure to allergens and irritants, such as using dust mite-proof pillow and mattress covers, and avoiding pets and tobacco smoke.*

*5. Monitor the patient's blood pressure and cholesterol levels to ensure appropriate management of her hypertension and dyslipidemia.*

***Surgical management:***

*If the patient's symptoms do not improve with medical management, the otolaryngologist may consider the following surgical interventions:*

*1. Functional endoscopic sinus surgery (FESS) to address the chronic rhinosinusitis by removing any obstructions and improving sinus drainage.*

*2. Inferior turbinate reduction surgery to decrease the size of the hypertrophied turbinates and improve nasal airflow. This can be done using various techniques such as radiofrequency ablation, coblation, or partial resection.*

*3. Septoplasty to correct the right septal deviation and improve nasal breathing. This procedure involves the surgical straightening of the nasal septum.*

*The decision to proceed with surgery would be based on the severity of the patient's symptoms, their response to medical management, and the potential risks and benefits of the procedures. The otolaryngologist would discuss these factors with the patient and involve them in the decision-making process.*

*It is essential for the patient to consult with a qualified healthcare professional to receive an accurate diagnosis and appropriate treatment recommendations based on their specific medical history and condition.*
